# Supplementary material for: Integrating humanoid robots with human musicians for synchronized musical performances
Source: PeerJ Comput Sci. 2025 Jan 3;11:e2632. doi: 10.7717/peerj-cs.2632 (PMC11784815; doi:10.7717/peerj-cs.2632)
Supplement: Supplemental Information 1 — Unified ROS framework for both Polaris and Oscar. [file peerj-cs-11-2632-s001.zip › Code/src/events/src/remote_control_server/web_client/html/index.html]

robot pos

POLY obstacles

RECT obstacles

walls

circle obstacles

marker


enter

submit

cancel

Map property:  


get map


map height


map width

event track bars

robot status:

status

Start


Reset

Click the input box below to control robot with keyboard

These buttons may also be used to control the robot

w: forward

s: backward

a: turn left

d: turn right

1: open door

2: turn tube

0: reset

Log:
