# Supplementary material for: Integrating humanoid robots with human musicians for synchronized musical performances
Source: PeerJ Comput Sci. 2025 Jan 3;11:e2632. doi: 10.7717/peerj-cs.2632 (PMC11784815; doi:10.7717/peerj-cs.2632)
Supplement: Supplemental Information 1 — Unified ROS framework for both Polaris and Oscar. [file peerj-cs-11-2632-s001.zip › Code/src/ROBOTIS-OP3-Tools/op3_web_setting_tool/html/index.html]

ROBOTIS OP3 Demo


# ROBOTIS OP3 parameter setting

- Demo
- Soccer
- Vision
- Action
- Camera

#### Mode

 Ready 
 Soccer 
 Action 
 Vision 

#### Demo Command

 Start 
 Stop

#### Ball detector

|  |  |  |
| --- | --- | --- |
| gaussian blur size |  |  |
| gaussian blur sigma |  |  |
| canny edge threshold |  |  |
| hough accum resolution |  |  |
| min circle distance |  |  |
| hough accum threshold |  |  |
| min radius |  |  |
| max radius |  |  |
| ellipse size |  |  |

|  |  |  |
| --- | --- | --- |
| Hue min |  |  |
| Hue max |  |  |
| Saturation min |  |  |
| Saturation max |  |  |
| Value min |  |  |
| Value max |  |  |

|  |  |
| --- | --- |
| Debug Image   Normal Image | Reset parameters |

#### Face tracker

#### Action

 Walking Ready 
 Sit Down 
 Stand Up 
 Thank you 
  
  
 Get up(Front) 
 Get up(Back) 
 Right Kick 
 Left Kick 
  
  
 Action Index : 

 Play Action

#### usb\_cam\_node

|  |  |  |
| --- | --- | --- |
| brightness |  |  |
| contrast |  |  |
| saturation |  |  |
| sharpness |  |  |
| gain |  |  |
| focus\_auto |  |  |
| focus\_absolute |  |  |
| exposure\_auto | auto(0) manual(1) shutter\_priority(2) aperture\_priority(3) |  |
| exposure\_absolute |  |  |
| white\_balance\_temperature\_auto |  |  |
| white\_balance\_temperature |  |  |

Reset parameters
