# Supplementary material for: Integrating humanoid robots with human musicians for synchronized musical performances
Source: PeerJ Comput Sci. 2025 Jan 3;11:e2632. doi: 10.7717/peerj-cs.2632 (PMC11784815; doi:10.7717/peerj-cs.2632)
Supplement: Supplemental Information 1 — Unified ROS framework for both Polaris and Oscar. [file peerj-cs-11-2632-s001.zip › Code/src/ROBOTIS-OP3-Tools/op3_web_setting_tool/html-li/index_good_for_test_1.html]

ROBOTIS OP3 Demo


# ROBOTIS OP3 parameter setting

#### Ball detector

|  |  |
| --- | --- |
| Debug Image  Normal Image | Reset parameters |
